# Enhanced Photocatalysis of Electrically Polarized Titania Nanosheets

**DOI:** 10.3390/nano14020171

**Published:** 2024-01-12

**Authors:** Tomoyuki Mihara, Kosuke Nozaki, Yasuyuki Kowaka, Mengtian Jiang, Kimihiro Yamashita, Hiroyuki Miura, Satoshi Ohara

**Affiliations:** 1 Graduate School of Medical and Dental Sciences, Tokyo Medical and Dental University, Bunkyo-ku, Tokyo 113-8549, Japan; t.mihara.fpro@tmd.ac.jp (T.M.); y.kowaka.fpro@tmd.ac.jp (Y.K.); jiang.mt.fpro@tmd.ac.jp (M.J.); yama-k.bcr@tmd.ac.jp (K.Y.); h.miura.fpro@tmd.ac.jp (H.M.); 2 New Industry Creation Hatchery Center, Tohoku University, Aoba, Sendai 980-8579, Japan; s.ohara@super-nano.com

**Keywords:** TiO_2_, surface charge, electrical polarization, photocatalytic activity, dye decolorization

## Abstract

Titania (TiO_2_) nanosheets are crystals with controlled, highly ordered structures that improve the functionality of conventional TiO_2_ nanoparticles. Various surface modification methods have been studied to enhance the effectiveness of these materials as photocatalysts. Surface modifications using electrical polarization have attracted considerable attention in recent years because they can improve the function of titania without changing its composition. However, the combination of facet engineering and electrical polarization has not been shown to improve the functionality of TiO_2_ nanosheets. In the present study, the dye-degradation performance of polarized TiO_2_ nanosheets was evaluated. TiO_2_ nanosheets with a F/Ti ratio of 0.3 were synthesized via a hydrothermal method. The crystal morphology and structure were evaluated using transmission electron microscopy and X-ray diffraction. Then, electrical polarization was performed under a DC electric field of 300 V at 300 °C. The polarized material was evaluated using thermally stimulated current measurements. A dye-degradation assay was performed using a methylene blue solution under ultraviolet irradiation. The polarized TiO_2_ nanosheets exhibited a dense surface charge and accelerated decolorization. These results indicate that electrical polarization can be used to enhance the photocatalytic activity of TiO_2_.

## 1. Introduction

Titania (TiO_2_) has been used in industrial applications as well as in the dental field as a denture cleaner and for tooth bleaching because it causes a reduction–oxidation (redox) reaction through photocatalytic activity on its surface [1]. Ultraviolet (UV) irradiation generates electrons and holes on the surface of titania, resulting in the generation of reactive oxygen species (ROS) such as superoxide anions (˙O_2_^−^) and hydroxyl radicals (˙OH) in the presence of water and oxygen [2]. These oxidizing substances can decompose organic dyes with a higher efficiency than common oxygen molecules [3]. However, the generated electrons and holes recombine within 50 ns, indicating that carriers have a longer lifetime on their surfaces [4].

The photocatalytic activity of titania has been improved using various methods, such as facet engineering [5,6], structure regulation [7], and metal doping [8]. These methods aim to enhance the photocatalytic activity by delaying electron–hole recombination. Especially enhanced photocatalysis has been obtained by designing compact heterojunctions [9]. We previously reported a highly ordered, facet-controlled, anatase-type titania nanosheet (NS) with several {001} planes and found that it had superior photocatalytic activity and antibacterial capability compared to conventional titania nanoparticles [10,11]. Titania NSs with side lengths of 29 nm were synthesized using ammonium hexafluorotitanate ((NH_4_)_2_TiF_6_) and titanium butoxide (Ti(OBu)_4_). Fluoride from ammonium hexafluorotitanate inhibits crystal growth of the {001} facet by adhering to its facet, which has a high surface energy. The {001} surface has also been reported to have a higher reactivity site against UV irradiation than the {101} surface, which causes redox reactions on both the {001} and {101} surfaces [12]. These results suggest that facet-engineered titania is crucial for the design of photocatalyst-containing biomaterials. However, decolorization was achieved through decomposition via photocatalytic activity as well as by adsorbing dyes on the titania surface [13].

Dye adsorption is typically regulated by physical forces such as van der Waals forces, hydrogen bonds, polarity, and dipole–dipole interactions [14]. The surface charge also plays a critical role in dye adsorption [15]. Electrical polarization has attracted considerable attention as a promising surface modification method [16,17,18]. We previously reported the surface modification of bioceramics, including hydroxyapatite, carbonated apatite, β-tricalcium phosphate (TCP), zirconia, bioglasses, and glass ceramics, through the electrical polarization process, which can induce surface charges without any doped elements or coating [19,20,21,22,23]. Although the strong ferroelectric polarization of TiO_2_ can be achieved through the conjunction of perovskite-based material layers [24], such as BaTiO_3_ and SrTiO_3_, for applications in the medical and dental fields, surface modifications without any other impurities are highly recommended.

Electrical polarization also regulates titania’s electrical dipole orientation under DC electric fields and a high temperature of approximately 300 to 400 °C and maintains its orientation at room temperature [25]. Electrically polarized titania has been reported to enhance osteoblast-like cell proliferation and differentiation and osteoconductivity [23,25,26]. In previous studies, polarized titania was fabricated using the micro-arc oxidation method, which incorporates calcium and phosphate ions. However, it is unclear whether the dipole orientation of titania without doped elements can be induced using electrical polarization. Concerning metal Ti-oxidated titania, we electrically polarized a thick titania layer on a titanium substrate. Bandyopadhyay et al. also reported that titania nanotubes on Ti can be polarized by electrothermal polarization and are supposed to be a novel biomaterial to promote in vivo osseointegration at an early stage in vivo [27]. It is still unclear whether titania powder can be electrically polarized.

The purpose of this study was to attempt the surface modification of NSs by electrical polarization and evaluate the dye-degradation capability of polarized titania NSs to clarify their functions. TiO_2_ NSs with a fluorine-to-titanium molar ratio of 0.3 were hydrothermally synthesized. The crystal structure was characterized using transmission electron microscopy (TEM) and X-ray diffraction (XRD). Subsequently, electrical polarization was performed, and the surface charges were evaluated using thermally stimulated current (TSDC) measurements. The dye-degradation performance of the polarized NS was evaluated using a methylene blue (MB) dye-degradation test. The null hypothesis was that electrical polarization does not enhance the decolorizing performance of TiO_2_.

## 2. Materials and Methods

### 2.1. Synthesis of Titania Nanosheets

The details of the fabrication process for the titania NSs have been described previously [10]. Briefly, titanium butoxide (Sigma-Aldrich, St. Louis, MO, USA) was added dropwise with stirring to 37% hydrochloric acid (FUJIFILM Wako Pure Chemical Corp., Osaka, Japan) and ammonium hexafluorotitanate (Sigma-Aldrich, St. Louis, MO, USA) at a F/Ti ratio of 0.3. The precursor was transferred to a high-pressure reaction vessel and hydrothermally synthesized at 180 °C for 6 h. The precipitates were sonicated thrice with methanol and twice with distilled water. The precipitates were then freeze-dried to obtain a titania NS using a freeze dryer (FDS-1000, Tokyo Rikakikai Co., Ltd., Tokyo, Japan). Titania nanoparticles (NPs, FUJIFILM Wako Pure Chemical Corp. Osaka, Japan) were used as control materials.

### 2.2. Electrical Polarization

**Figure 1:**
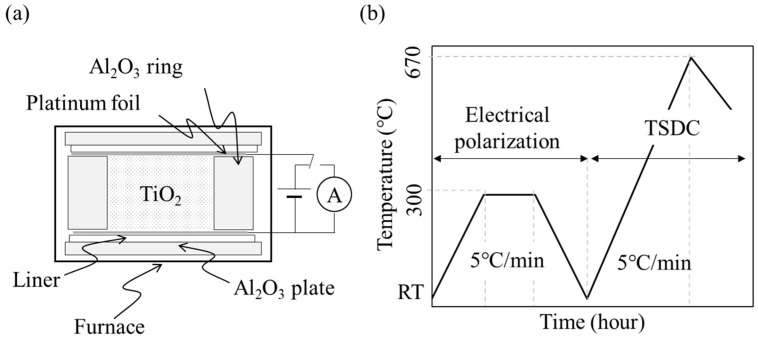
(**a**) Schematic diagram of sample preparation for electrical polarization. For the thermally stimulated depolarization current (TSDC) measurement, the electrode was connected to a galvanometer. (**b**) Temperature program for electrical polarization and TSDC measurement procedure.

NS and NP powders were poured into an alumina ring with an inner diameter of 10 mm and a depth of 2 mm, sandwiched between platinum foil electrodes, and then electrically polarized under a DC electric field at 300 V/mm and 300 °C for 1 h (P-NS, P-NP) (Figure 1). As the control group, NSs and NPs were subjected to the same process of heating at 300 °C under 0 V/mm (H-NS, H-NP).

### 2.3. Characterization

The crystal morphology and structure of each sample were observed using TEM (H-7100/XR81, Hitachi Ltd., Tokyo, Japan) (JEM-1400Flash, JEOL Ltd., Tokyo, Japan). In addition, the phase composition of the sample surface was analyzed in the 2θ range of 20° to 80° using XRD (D8 advance, Bruker AXS GmbH, Karlsruhe, Germany).

Polarized and non-polarized samples were evaluated using TSDC at a temperature increase rate of 5.0 °C/min from room temperature to 670 °C in air after polarization with a galvanometer (6514/J, Tektronix Inc., Tokyo, Japan) (Figure 1) [20,21,22,26,27]. The stored charge (Q) was calculated from the TSDC spectra using the following equation:

$$Q=\frac{1}{\beta }\;\int J\left(T\right)dT$$

where *Q* is the stored charge (C/cm^2^), *β* is the ramp rate of the temperature (K/s), and *J(T)* is the depolarization current density (A/cm*^2^*).

The optical properties of the heated and polarized samples were characterized using UV-visible (UV-vis) spectrophotometry (Jasco V-550, JASCO International Co. Ltd., Tokyo, Japan). The optical bandgap energies (Eg) of the polarized and non-polarized sample were calculated using Tauc plots, as previously described [28].

### 2.4. Dye-Degradation Assay

**Figure 2:**
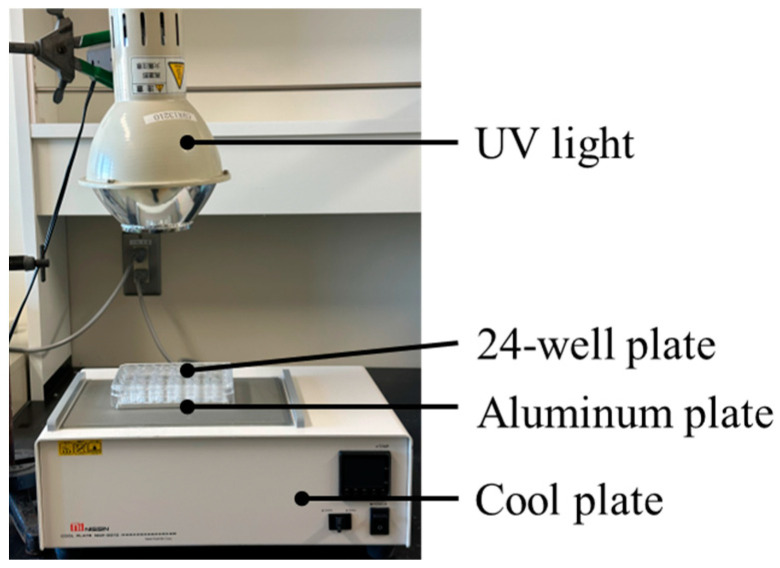
Experimental setup for dye-degradation assay. The temperature of the dye solution in a 24-well plate was regulated by a cool plate via an aluminum plate.

The dye-degradation test using the redox reaction was conducted using a 0.3 mM MB solution. Each sample was mixed with MB at a concentration of 10 mg/mL. The mixed samples were irradiated at 20 °C (NCP2215, Nissinrika Corp., Tokyo, Japan) for 1, 10, 30, 60, 120, 180, 240, or 480 min under UV light (HL100G, Sen special light Source, SEN LIGHTS CORP., Osaka, Japan) at 2.5 mW/cm^2^ (Figure 2). The samples were centrifuged (20,000× *g*, 10 min), the supernatant was diluted 10-fold, and the absorbance was measured at 630 nm using a microplate reader (Model680, Bio-Rad Laboratories Inc., Hercules, Berkeley, CA, USA). For the non-irradiated group, the absorbance was measured in the same manner after UV irradiation under the same conditions, shielded with aluminum foil. After obtaining the absorbance at each time point, the rate constant of MB degradation was calculated using the following equation:

$$\ln\frac{A_{0}}{A_{t}}=k_{a}t$$

where *k_a_* is a rate constant, *A_t_* is the absorbance of the MB solution at each time, and *A*_0_ is the absorbance after 1 min.

### 2.5. Measurement of ROS

To measure the ROS generated from the titania samples (P-NS, P-NP, H-NS, and H-NP) and their reactions under UV irradiation, disodium terephthalate (DTA, Tokyo Kasei Kogyo, Tokyo, Japan) and nitro blue tetrazolium (NBT, Tokyo Kasei Kogyo, Tokyo, Japan) assays were performed. NBT and DTA react with superoxide anions and hydroxyl radicals and convert them to formazan and 2-hydroxy terephthalic acid (HTA), respectively [10,29].

The NBT solution was mixed with 4 mg/mL of TiO_2_ and irradiated with UV light at 2.5 mW/cm^2^ for 2 h. After irradiation, the solution was centrifuged at 13,000 rpm for 10 min, and the supernatant was discarded. The precipitates were dissolved in 100 μL of dimethyl sulfoxide (DMSO, Fujifilm Wako Pure Chemicals Corporation, Osaka, Japan) and stirred for 10 min. The absorbance was measured at 570 nm using a spectrophotometer.

Titania was added to a 20 mM DTA solution and UV-irradiated under the same conditions as those used for the NBT assay. The reaction solution was centrifuged, and the supernatant was collected. The fluorescence intensity of the supernatant was measured using a fluorescence microplate reader (Wallac Arvo MX; Perkin Elmer Co., Ltd., Waltham, MA, USA).

As previously reported [10], the absorbance and fluorescence intensity were converted to the concentrations of superoxide anions and hydroxyl radicals, respectively [30,31]. Briefly, a reaction mixture containing riboflavin, methionine, and NBT in potassium phosphate buffer was incubated for 1 h at room temperature. The purple precipitates were dissolved in DMSO and diluted up to the representative concentrations.

Then, 2-hydroxy terephthalic acid (2-HTA) was dissolved in distilled water up to the representative concentrations. The concentration of the hydroxyl radical can be estimated using the following reaction:

NaTA+˙OH→2-HTA

Standard curves were constructed using representative concentrations of formazan and 2-HTA.

### 2.6. Statistical Analysis

A one-way analysis of variance followed by the Dunnet T3 test was used for statistical processing using statistical software (IBM SPSS Statistics v27.0; IBM Corp, New York, NY, USA), and the results with a *p*-value of 0.05 or less were considered statistically significant.

## 3. Results

### 3.1. Crystal Morphology and Structure of Polarized Titania

**Figure 3:**
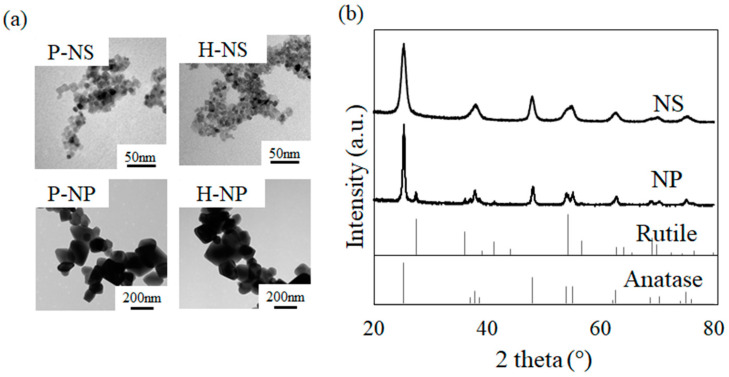
Characterization of polarized (P-NS, P-NP) and non-polarized (H-NS, H-NP) titania. (**a**) TEM images of titania; (**b**) XRD patterns of NSs and NPs.

The TEM images of the samples are shown in Figure 3a. The P-NS and H-NS had a slightly flattened crystal structure with an average side length, width, and thickness of 6.3 nm, 6.3 nm, and 4.9 nm, respectively. When comparing P-NS with H-NS, there was no difference in their morphology. In contrast to the NSs, P-NP and H-NP had a rounded rectangular crystal structure, but an irregular morphology with a large difference between the small (approximately 80 nm) and large (approximately 450 nm) crystals. When comparing P-NP with H-NP (Figure 3a), no differences in size or surface structure were observed. The crystal morphology was not changed by the electrical polarization treatment. The XRD patterns of the NSs and titania NPs are shown in Figure 3b. The spectrum shows that the NS samples were anatase type, whereas the NP samples were anatase and rutile composites.

### 3.2. Surface Charges and Optical Bandgap

**Figure 4:**
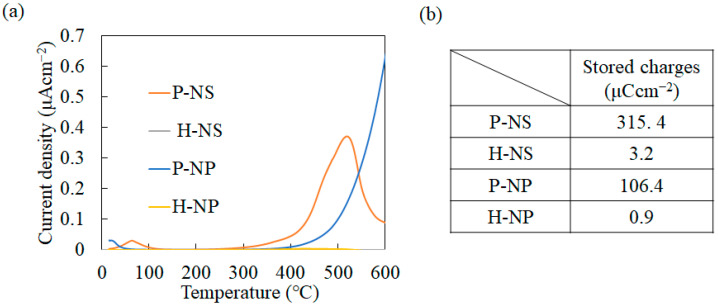
(**a**) Thermally stimulated depolarization current (TSDC) spectra of P-NS, H-NS, P-NP, and H-NP; (**b**) stored charges of P-NS, H-NS, P-NP, and H-NP. The spectra were cut off and calculated based on 540 °C.

The surface charges of polarized titania were analyzed using TSDC measurements. The representative TSDC spectra are shown in Figure 3a. P-NS showed peaks at 68 °C and 519 °C, while P-NP showed peaks at 25 °C and divergent waveforms starting at 300 °C. No peaks were observed for the H-NS or H-NP. The accumulated stored charge was calculated from the TSDC spectra via integration (Figure 4b). The accumulated charge of P-NS was 315.4 μCcm^−2^, and that of P-NP was 106.4 μCcm^−2^, whereas those of H-NS and H-NP were 3.2 μCcm^−2^ and 0.9 μCcm^−2^, respectively.

**Figure 5:**
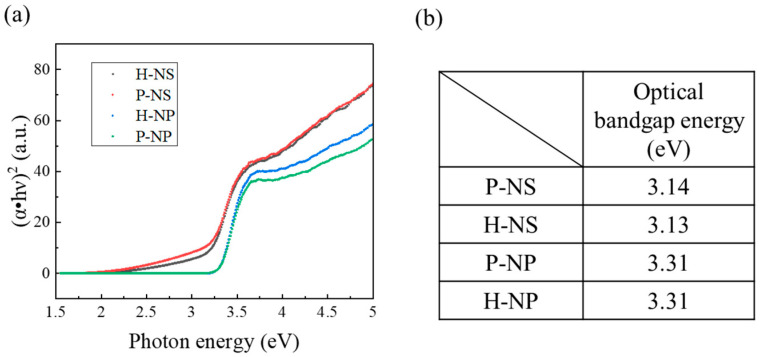
(**a**) Tauc plots and (**b**) optical bandgap energy of heated and polarized TiO_2_ NSs (H-NS and P-NS) and NPs (H-NP and P-NP).

Tauc plots were drawn from the UV-vis absorbance results, and the optical bandgaps were determined (Figure 5). The optical bandgaps of H-NS, P-NS, H-NP, and P-NP were 3.13, 3.14, 3.31, and 3.31 eV, respectively.

### 3.3. Effects of Electrical Polarization on Photocatalytic Properties of Titania Nanosheet

#### 3.3.1. Production of Hydroxyl Radical and Superoxide Anion under UV Irradiation

**Figure 6:**
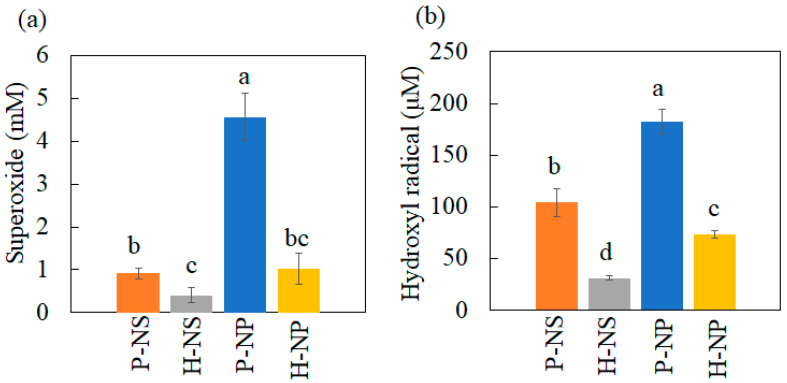
ROS production under UV irradiation. (**a**) Superoxide anion production and (**b**) hydroxyl radical production. The different letters show significant differences at α = 0.05.

The generation of superoxide anions and hydroxyl radicals was evaluated using nitro blue tetrazolium and DTA assays. Because the amounts of converted formazan and 2-HTA are proportional to the amount of ROS, the concentration of ROS was calculated from the standard curves, as shown in Figure 6. P-NP showed the highest efficiency in generating superoxide and hydroxyl radicals. The polarized titania also generated more ROS than non-polarized titania.

#### 3.3.2. Decolorization Experimental Results

**Figure 7:**
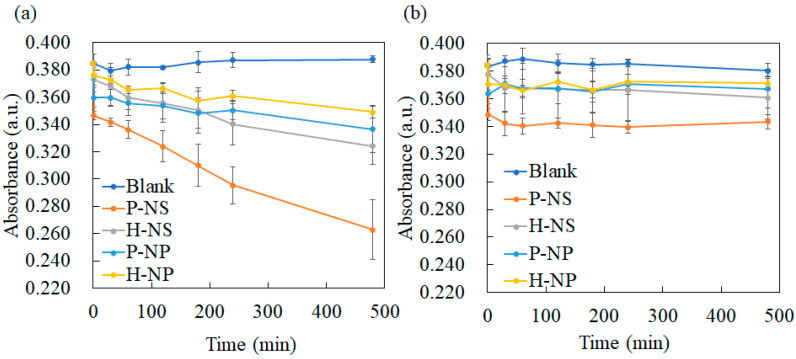

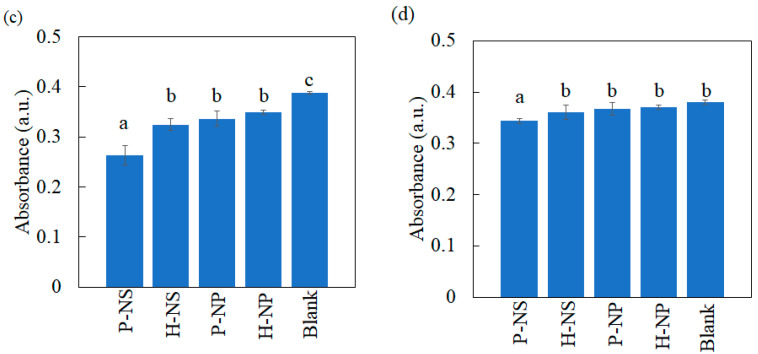
Time course plot of the MB decolorization reaction using the prepared titania samples (**a**) under UV irradiation and (**b**) in the dark. Multiple comparisons of MB absorbance after 480 min (**c**) under UV irradiation and (**d**) in the dark. The different letters show significant differences at α = 0.05.

**Figure 8:**
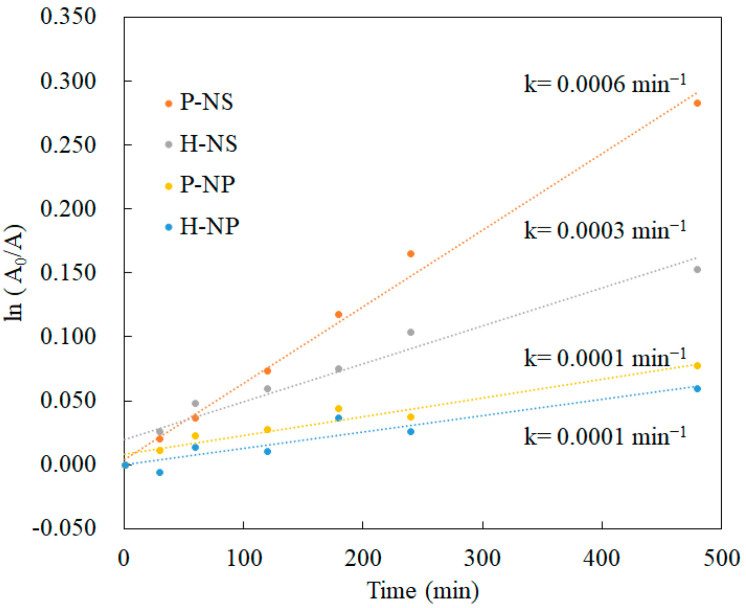
Reaction rate of ln(A_0_/A) of MB under UV irradiation.

The decolorization of the MB solution with titania without UV irradiation was evaluated at regular intervals. Under UV irradiation, MB decomposed in a time-dependent manner (Figure 7a). P-NS showed the highest dye-degradation efficiency after 480 min (Figure 7a). Electrical polarization enhanced dye degradation in the NS group, but no effect was observed in the NP group. The decolorization of MB without UV irradiation showed no time-dependent changes after 30 min (Figure 7b). However, a significant difference was observed after 30 min (Figure 7b). Polarized titania showed significantly enhanced decolorization compared to non-polarized titania. The calculated rate constant of P-NS was higher than those of H-NS, H-NP, and P-NP (Figure 8). The electrical polarization of the NSs enhanced the calculated rate constant.

## 4. Discussion

This is the first study to regulate the surface charges of TiO_2_ NPs and NSs using the electrical polarization method. We showed enhanced dye-degradation activity of polarized TiO_2_ NSs. To modify the degradation performance of TiO_2_, the crystal growth was regulated by supplying F ions during the hydrothermal synthesis using ammonium hexafluorotitanate and titanium butoxide as starting materials with a F/Ti ratio of 0.3. Anatase-type TiO_2_ NSs as small as 6.3 nm were obtained. The TiO_2_ powder was then electrically polarized by a DC electric field at 300 V/mm and 300 °C for 1 h to control the surface charges. The TSDC results showed that polarized TiO_2_ NSs were successfully prepared. Accelerated dye degradation was achieved by P-NS, but not by polarized P-NP. However, superoxide anion and hydroxyl radical generation, detected using NBT and DTA assays, was enhanced by both P-NS and P-NP. From these results, the null hypothesis, which states that electrical polarization does not enhance the decolorizing performance of TiO_2_, was rejected, as P-NS accelerated MB degradation compared to H-NS.

Titania NSs have been reported to have high photocatalytic activity because of the heterojunctions of the {101}/{001} plane, and they are considered an alternative for titania nanoparticles [32]. We previously synthesized titania NSs with a side length of 6.3 to 445 nm and clarified that the best photocatalytic activity was achieved by titania NSs with a side length of 6.3 nm [28]. In this study, a titania NS with a side length of 6.3 nm was used as a representative substrate for electrical polarization because the single-nanometer-sized titania NS showed the highest dye-degradation performance compared to the other NSs and NPs.

Dipole formation in a crystal structure originates from the orientation of the positive and negative ions. Electrical polarization enables the reorientation of ions at room temperature to be fixed by a constant DC electric field from high to low temperatures without substituting ions. TSDC measurements can evaluate the surface charges as an electric current induced by the relaxation of dipoles through a heating process. The surface charges of TiO_2_ NPs and NSs were estimated to be induced by the reorientation of oxygen vacancies and Ti^3+^ because regularly synthesized TiO_2_ contains 1% oxygen vacancies [33]. Furthermore, the TiO_2_ NSs contained F^-^, not only at the surface, but also in the lattice, substituting for O^2−^ and inducing the reduction of Ti^4+^ to Ti^3+^ to compensate for the charges [34]. Hence, P-NS dipoles may be induced by reorienting the oxygen vacancy and F^−^.

In the decolorization experiment, P-NS showed the strongest decolorizing power after 480 min compared with H-NS, P-NP, and H-NP. These results agree with the trend in our previous report showing the better decolorizing capability of NSs compared to that of NPs. Furthermore, electrical polarization accelerated the decolorization of MB by P-NS. The TSDC measurements confirmed that the surface charges of P-NS and P-NP were successfully induced by electrical polarization. Azeez et al. reported that the MB adsorption by TiO_2_ NPs was enhanced by the higher surface charges originating from the rich hydroxyl groups [13]. However, this study showed that the adsorption of the MB dye was also enhanced by electrical polarization, inducing surface charges without adding any elements to the surfaces. This study also demonstrated that polarized NSs and NPs induced the production of more superoxide anions and hydroxyl radicals under UV irradiation compared to the non-polarized samples. Although ROS generation can be enhanced by accelerated redox reactions on the surface of TiO_2_ by UV irradiation, the optical bandgap energy results indicated that electrical polarization had no influence on the reactivity against UV irradiation. ROS are usually detected by indirect methods using scavengers such as DTA and NBT because of the rapid reactions and decomposition during UV irradiation [2]. This indicates that the polarized TiO_2_ accelerated the reaction of DTA and NBT through redox reactions, following the enhanced adsorption of scavengers.

Despite the greater generation of ROS by P-NP and H-NP, the decolorizing effects of P-NS and H-NS were greater than those of P-NP and H-NP. This is because the formation of ROS has been reported to be enhanced by the mixed phase of rutile and anatase [35]. The commercially available NPs used in this study showed a mixed phase of anatase and rutile phases, whereas the NSs showed a single anatase phase. A previous study indicated that the adsorption of MB was enhanced by TiO_2_ NSs compared to that by TiO_2_ NPs [36], which resulted in accelerated dye degradation by the P-NS.

From the above results, the dye-degradation process by polarized TiO_2_ NSs under aqueous conditions was proposed as follows. The adsorption of efficient photons by TiO_2_ NSs generated electrons and holes, which resulted in the generation of superoxide anions and hydroxyl radicals. The polarized TiO_2_ NSs absorbed more MB dye compared to the polarized TiO_2_ NPs, heated TiO_2_ NSs, or heated TiO_2_ NPs. Oxidation by the generated ROS and direct oxidation by the reaction with holes degraded the MB solution.

## 5. Conclusions

A facile preparation of TiO_2_ NSs using a hydrothermal synthesis with a fluorine/titanium ratio of 0.3 and the modification of surface charges by electrical polarization methods was reported. The synthesized TiO_2_ NSs formed a flattened crystal structure with an average side length and thickness of 6.3 nm and 4.9 nm, respectively. TSDC measurements showed a dense surface charge of P-NS and P-NP without changing their morphology or optical properties. The MB degradation assay using P-NS showed an increase in the dye-degradation rate and adsorption efficiency. Furthermore, P-NS and P-NP accelerated the generation of hydroxyl and superoxide anions, respectively. This simple and robust technique can be applied to various ceramic nanocatalysts to manipulate their surface charge and enhance their photocatalytic activity. Considering the enhanced photocatalytic activity of polarized TiO_2_ NSs, the band structure of heated and polarized TiO_2_ will be further investigated to clarify the mechanisms of enhanced dye degradation.

## Data Availability

Data is contained within the article.
